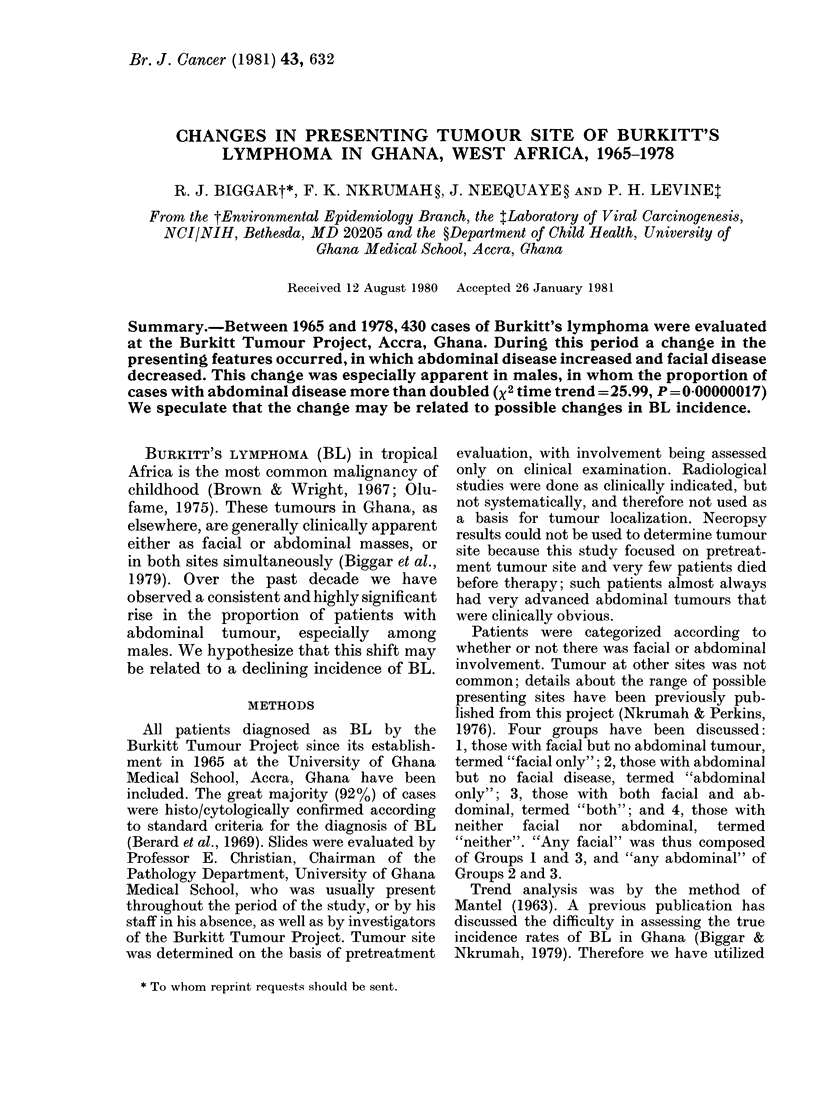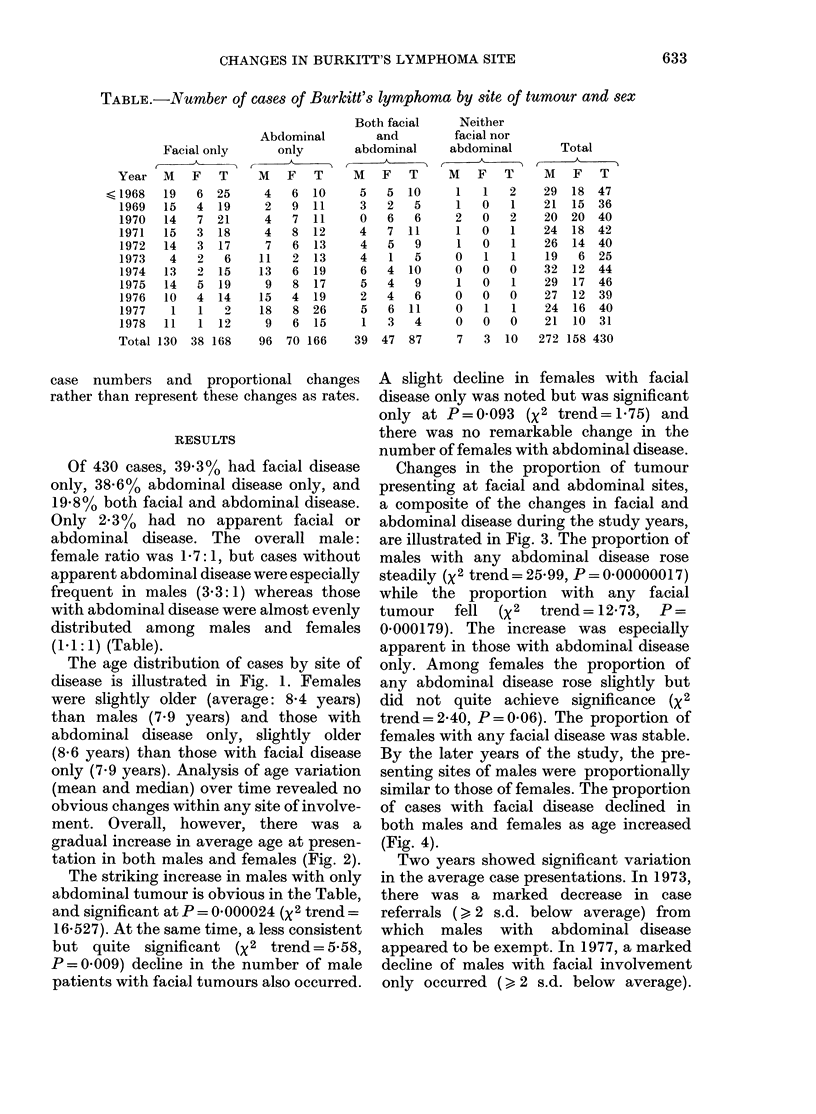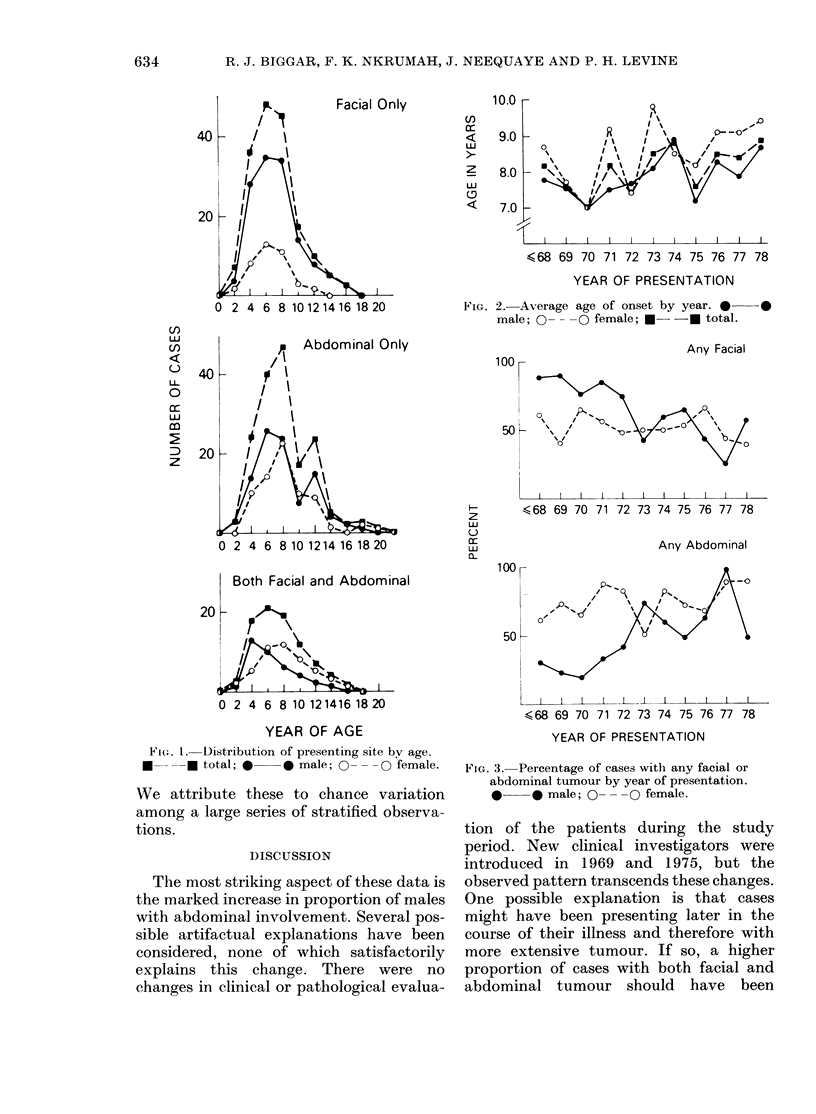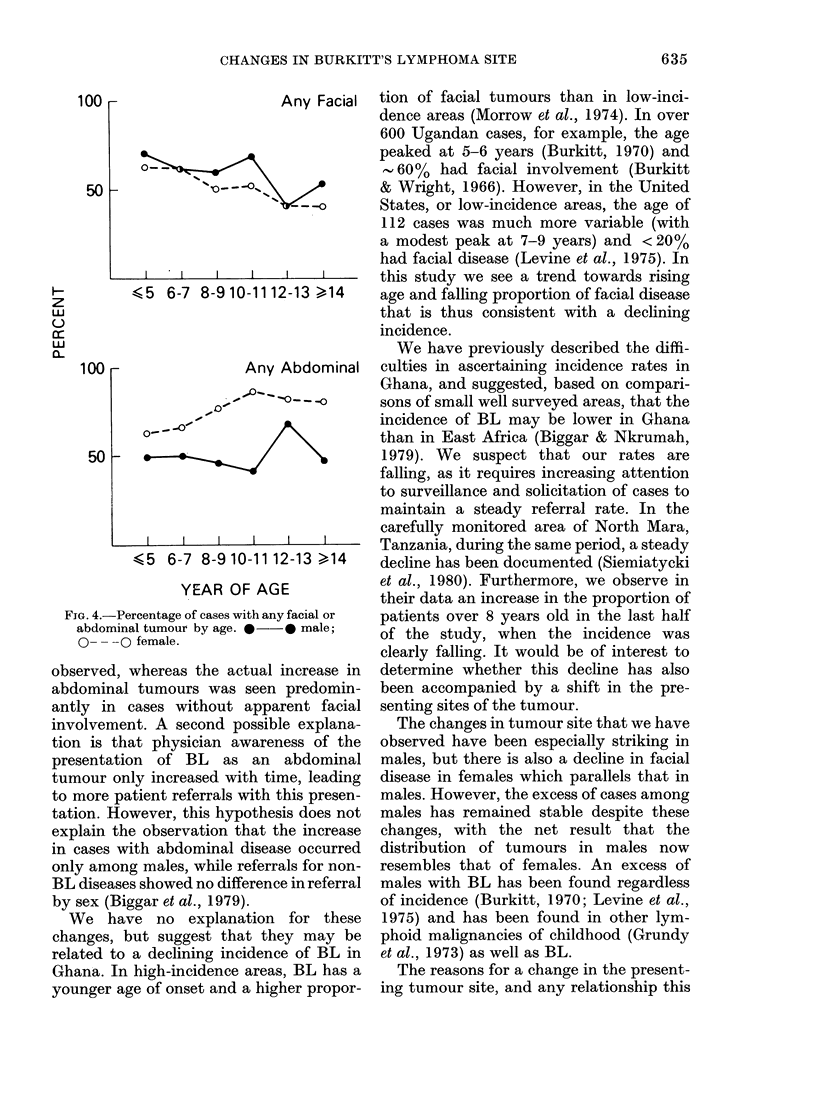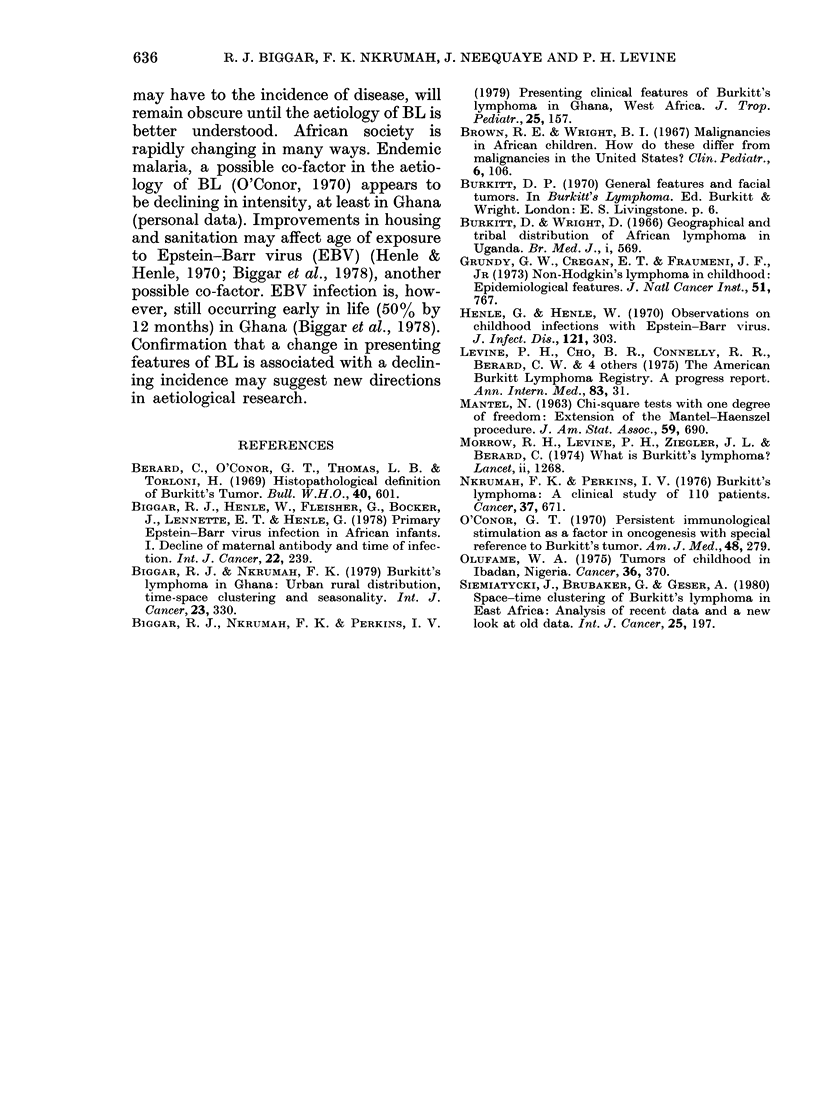# Changes in presenting tumour site of Burkitt's lymphoma in Ghana, West Africa, 1965-1978.

**DOI:** 10.1038/bjc.1981.93

**Published:** 1981-05

**Authors:** R. J. Biggar, F. K. Nkrumah, J. Neequaye, P. H. Levine

## Abstract

Between 1965 and 1978, 430 cases of Burkitt's lymphoma were evaluated at the Burkitt Tumour Project, Accra, Ghana. During this period a change in the presenting features occurred, in which abdominal disease increased and facial disease decreased. This change was especially apparent in males, in whom the proportion of cases with abdominal disease more than doubled (chi 2 time trend = 25.99, P = 0.00000017) We speculate that the change may be related to possible changes in BL incidence.


*Br. J. Cancer* (1981) **43**, 632

### CHANGES IN PRESENTING TUMOUR SITE OF BURKITT'S LYMPHOMA IN GHANA, WEST AFRICA, 1965–1978

R. J. BIGGAR†*, F. K. NKRUMAH§, J. NEEQUAYE§ AND P. H. LEVINE‡

*From the †Environmental Epidemiology Branch, the ‡Laboratory of Viral Carcinogenesis, NCI/NIH, Bethesda, MD 20205 and the §Department of Child Health, University of Ghana Medical School, Accra, Ghana*

Received 12 August 1980 Accepted 26 January 1981

**Summary.—Between 1965 and 1978, 430 cases of Burkitt's lymphoma were evaluated at the Burkitt Tumour Project, Accra, Ghana. During this period a change in the presenting features occurred, in which abdominal disease increased and facial disease decreased. This change was especially apparent in males, in whom the proportion of cases with abdominal disease more than doubled ($\chi^2$ time trend = 25.99, $P = 0.00000017$) We speculate that the change may be related to possible changes in BL incidence.**

BURKITT'S LYMPHOMA (BL) in tropical Africa is the most common malignancy of childhood (Brown & Wright, 1967; Olufame, 1975). These tumours in Ghana, as elsewhere, are generally clinically apparent either as facial or abdominal masses, or in both sites simultaneously (Biggar *et al.*, 1979). Over the past decade we have observed a consistent and highly significant rise in the proportion of patients with abdominal tumour, especially among males. We hypothesize that this shift may be related to a declining incidence of BL.

### METHODS

All patients diagnosed as BL by the Burkitt Tumour Project since its establishment in 1965 at the University of Ghana Medical School, Accra, Ghana have been included. The great majority (92%) of cases were histo/cytologically confirmed according to standard criteria for the diagnosis of BL (Berard *et al.*, 1969). Slides were evaluated by Professor E. Christian, Chairman of the Pathology Department, University of Ghana Medical School, who was usually present throughout the period of the study, or by his staff in his absence, as well as by investigators of the Burkitt Tumour Project. Tumour site was determined on the basis of pretreatment evaluation, with involvement being assessed only on clinical examination. Radiological studies were done as clinically indicated, but not systematically, and therefore not used as a basis for tumour localization. Necropsy results could not be used to determine tumour site because this study focused on pretreatment tumour site and very few patients died before therapy; such patients almost always had very advanced abdominal tumours that were clinically obvious.

Patients were categorized according to whether or not there was facial or abdominal involvement. Tumour at other sites was not common; details about the range of possible presenting sites have been previously published from this project (Nkrumah & Perkins, 1976). Four groups have been discussed: 1, those with facial but no abdominal tumour, termed "facial only"; 2, those with abdominal but no facial disease, termed "abdominal only"; 3, those with both facial and abdominal, termed "both"; and 4, those with neither facial nor abdominal, termed "neither". "Any facial" was thus composed of Groups 1 and 3, and "any abdominal" of Groups 2 and 3.

Trend analysis was by the method of Mantel (1963). A previous publication has discussed the difficulty in assessing the true incidence rates of BL in Ghana (Biggar & Nkrumah, 1979). Therefore we have utilized

* To whom reprint requests should be sent.

TABLE.—*Number of cases of Burkitt's lymphoma by site of tumour and sex*

| Year | Facial only | | | Abdominal only | | | Both facial and abdominal | | | Neither facial nor abdominal | | | Total | | |
|---|---|---|---|---|---|---|---|---|---|---|---|---|---|---|---|
| | M | F | T | M | F | T | M | F | T | M | F | T | M | F | T |
| ≤ 1968 | 19 | 6 | 25 | 4 | 6 | 10 | 5 | 5 | 10 | 1 | 1 | 2 | 29 | 18 | 47 |
| 1969 | 15 | 4 | 19 | 2 | 9 | 11 | 3 | 2 | 5 | 1 | 0 | 1 | 21 | 15 | 36 |
| 1970 | 14 | 7 | 21 | 4 | 7 | 11 | 0 | 6 | 6 | 2 | 0 | 2 | 20 | 20 | 40 |
| 1971 | 15 | 3 | 18 | 4 | 8 | 12 | 4 | 7 | 11 | 1 | 0 | 1 | 24 | 18 | 42 |
| 1972 | 14 | 3 | 17 | 7 | 6 | 13 | 4 | 5 | 9 | 1 | 0 | 1 | 26 | 14 | 40 |
| 1973 | 4 | 2 | 6 | 11 | 2 | 13 | 4 | 1 | 5 | 0 | 1 | 1 | 19 | 6 | 25 |
| 1974 | 13 | 2 | 15 | 13 | 6 | 19 | 6 | 4 | 10 | 0 | 0 | 0 | 32 | 12 | 44 |
| 1975 | 14 | 5 | 19 | 9 | 8 | 17 | 5 | 4 | 9 | 1 | 0 | 1 | 29 | 17 | 46 |
| 1976 | 10 | 4 | 14 | 15 | 4 | 19 | 2 | 4 | 6 | 0 | 0 | 0 | 27 | 12 | 39 |
| 1977 | 1 | 1 | 2 | 18 | 8 | 26 | 5 | 6 | 11 | 0 | 1 | 1 | 24 | 16 | 40 |
| 1978 | 11 | 1 | 12 | 9 | 6 | 15 | 1 | 3 | 4 | 0 | 0 | 0 | 21 | 10 | 31 |
| Total | 130 | 38 | 168 | 96 | 70 | 166 | 39 | 47 | 87 | 7 | 3 | 10 | 272 | 158 | 430 |

case numbers and proportional changes rather than represent these changes as rates.

### RESULTS

Of 430 cases, 39·3% had facial disease only, 38·6% abdominal disease only, and 19·8% both facial and abdominal disease. Only 2·3% had no apparent facial or abdominal disease. The overall male: female ratio was 1·7:1, but cases without apparent abdominal disease were especially frequent in males (3·3:1) whereas those with abdominal disease were almost evenly distributed among males and females (1·1:1) (Table).

The age distribution of cases by site of disease is illustrated in Fig. 1. Females were slightly older (average: 8·4 years) than males (7·9 years) and those with abdominal disease only, slightly older (8·6 years) than those with facial disease only (7·9 years). Analysis of age variation (mean and median) over time revealed no obvious changes within any site of involvement. Overall, however, there was a gradual increase in average age at presentation in both males and females (Fig. 2).

The striking increase in males with only abdominal tumour is obvious in the Table, and significant at $P = 0{\cdot}000024$ ($\chi^2$ trend = 16·527). At the same time, a less consistent but quite significant ($\chi^2$ trend = 5·58, $P = 0{\cdot}009$) decline in the number of male patients with facial tumours also occurred. A slight decline in females with facial disease only was noted but was significant only at $P = 0{\cdot}093$ ($\chi^2$ trend = 1·75) and there was no remarkable change in the number of females with abdominal disease.

Changes in the proportion of tumour presenting at facial and abdominal sites, a composite of the changes in facial and abdominal disease during the study years, are illustrated in Fig. 3. The proportion of males with any abdominal disease rose steadily ($\chi^2$ trend = 25·99, $P = 0{\cdot}00000017$) while the proportion with any facial tumour fell ($\chi^2$ trend = 12·73, $P = 0{\cdot}000179$). The increase was especially apparent in those with abdominal disease only. Among females the proportion of any abdominal disease rose slightly but did not quite achieve significance ($\chi^2$ trend = 2·40, $P = 0{\cdot}06$). The proportion of females with any facial disease was stable. By the later years of the study, the presenting sites of males were proportionally similar to those of females. The proportion of cases with facial disease declined in both males and females as age increased (Fig. 4).

Two years showed significant variation in the average case presentations. In 1973, there was a marked decrease in case referrals (≥ 2 s.d. below average) from which males with abdominal disease appeared to be exempt. In 1977, a marked decline of males with facial involvement only occurred (≥ 2 s.d. below average).

FIG. 1.—Distribution of presenting site by age. ■— —■ total; ●——● male; ○- - -○ female.

FIG. 2.—Average age of onset by year. ●——● male; ○- - -○ female; ■— —■ total.

FIG. 3.—Percentage of cases with any facial or abdominal tumour by year of presentation. ●——● male; ○- - -○ female.

We attribute these to chance variation among a large series of stratified observations.

### DISCUSSION

The most striking aspect of these data is the marked increase in proportion of males with abdominal involvement. Several possible artifactual explanations have been considered, none of which satisfactorily explains this change. There were no changes in clinical or pathological evaluation of the patients during the study period. New clinical investigators were introduced in 1969 and 1975, but the observed pattern transcends these changes. One possible explanation is that cases might have been presenting later in the course of their illness and therefore with more extensive tumour. If so, a higher proportion of cases with both facial and abdominal tumour should have been

FIG. 4.—Percentage of cases with any facial or abdominal tumour by age. ●——● male; ○- - -○ female.

observed, whereas the actual increase in abdominal tumours was seen predominantly in cases without apparent facial involvement. A second possible explanation is that physician awareness of the presentation of BL as an abdominal tumour only increased with time, leading to more patient referrals with this presentation. However, this hypothesis does not explain the observation that the increase in cases with abdominal disease occurred only among males, while referrals for non-BL diseases showed no difference in referral by sex (Biggar *et al.*, 1979).

We have no explanation for these changes, but suggest that they may be related to a declining incidence of BL in Ghana. In high-incidence areas, BL has a younger age of onset and a higher proportion of facial tumours than in low-incidence areas (Morrow *et al.*, 1974). In over 600 Ugandan cases, for example, the age peaked at 5–6 years (Burkitt, 1970) and ~60% had facial involvement (Burkitt & Wright, 1966). However, in the United States, or low-incidence areas, the age of 112 cases was much more variable (with a modest peak at 7–9 years) and <20% had facial disease (Levine *et al.*, 1975). In this study we see a trend towards rising age and falling proportion of facial disease that is thus consistent with a declining incidence.

We have previously described the difficulties in ascertaining incidence rates in Ghana, and suggested, based on comparisons of small well surveyed areas, that the incidence of BL may be lower in Ghana than in East Africa (Biggar & Nkrumah, 1979). We suspect that our rates are falling, as it requires increasing attention to surveillance and solicitation of cases to maintain a steady referral rate. In the carefully monitored area of North Mara, Tanzania, during the same period, a steady decline has been documented (Siemiatycki *et al.*, 1980). Furthermore, we observe in their data an increase in the proportion of patients over 8 years old in the last half of the study, when the incidence was clearly falling. It would be of interest to determine whether this decline has also been accompanied by a shift in the presenting sites of the tumour.

The changes in tumour site that we have observed have been especially striking in males, but there is also a decline in facial disease in females which parallels that in males. However, the excess of cases among males has remained stable despite these changes, with the net result that the distribution of tumours in males now resembles that of females. An excess of males with BL has been found regardless of incidence (Burkitt, 1970; Levine *et al.*, 1975) and has been found in other lymphoid malignancies of childhood (Grundy *et al.*, 1973) as well as BL.

The reasons for a change in the presenting tumour site, and any relationship this may have to the incidence of disease, will remain obscure until the aetiology of BL is better understood. African society is rapidly changing in many ways. Endemic malaria, a possible co-factor in the aetiology of BL (O'Conor, 1970) appears to be declining in intensity, at least in Ghana (personal data). Improvements in housing and sanitation may affect age of exposure to Epstein–Barr virus (EBV) (Henle & Henle, 1970; Biggar *et al.*, 1978), another possible co-factor. EBV infection is, however, still occurring early in life (50% by 12 months) in Ghana (Biggar *et al.*, 1978). Confirmation that a change in presenting features of BL is associated with a declining incidence may suggest new directions in aetiological research.

### REFERENCES

Berard, C., O'Conor, G. T., Thomas, L. B. & Torloni, H. (1969) Histopathological definition of Burkitt's Tumor. *Bull. W.H.O.*, **40**, 601.

Biggar, R. J., Henle, W., Fleisher, G., Bocker, J., Lennette, E. T. & Henle, G. (1978) Primary Epstein–Barr virus infection in African infants. I. Decline of maternal antibody and time of infection. *Int. J. Cancer*, **22**, 239.

Biggar, R. J. & Nkrumah, F. K. (1979) Burkitt's lymphoma in Ghana: Urban rural distribution, time-space clustering and seasonality. *Int. J. Cancer*, **23**, 330.

Biggar, R. J., Nkrumah, F. K. & Perkins, I. V. (1979) Presenting clinical features of Burkitt's lymphoma in Ghana, West Africa. *J. Trop. Pediatr.*, **25**, 157.

Brown, R. E. & Wright, B. I. (1967) Malignancies in African children. How do these differ from malignancies in the United States? *Clin. Pediatr.*, **6**, 106.

Burkitt, D. P. (1970) General features and facial tumors. In *Burkitt's Lymphoma*. Ed. Burkitt & Wright. London: E. S. Livingstone. p. 6.

Burkitt, D. & Wright, D. (1966) Geographical and tribal distribution of African lymphoma in Uganda. *Br. Med. J.*, i, 569.

Grundy, G. W., Cregan, E. T. & Fraumeni, J. F., Jr (1973) Non-Hodgkin's lymphoma in childhood: Epidemiological features. *J. Natl Cancer Inst.*, **51**, 767.

Henle, G. & Henle, W. (1970) Observations on childhood infections with Epstein–Barr virus. *J. Infect. Dis.*, **121**, 303.

Levine, P. H., Cho, B. R., Connelly, R. R., Berard, C. W. & 4 others (1975) The American Burkitt Lymphoma Registry. A progress report. *Ann. Intern. Med.*, **83**, 31.

Mantel, N. (1963) Chi-square tests with one degree of freedom: Extension of the Mantel–Haenszel procedure. *J. Am. Stat. Assoc.*, **59**, 690.

Morrow, R. H., Levine, P. H., Ziegler, J. L. & Berard, C. (1974) What is Burkitt's lymphoma? *Lancet*, ii, 1268.

Nkrumah, F. K. & Perkins, I. V. (1976) Burkitt's lymphoma: A clinical study of 110 patients. *Cancer*, **37**, 671.

O'Conor, G. T. (1970) Persistent immunological stimulation as a factor in oncogenesis with special reference to Burkitt's tumor. *Am. J. Med.*, **48**, 279.

Olufame, W. A. (1975) Tumors of childhood in Ibadan, Nigeria. *Cancer*, **36**, 370.

Siemiatycki, J., Brubaker, G. & Geser, A. (1980) Space–time clustering of Burkitt's lymphoma in East Africa: Analysis of recent data and a new look at old data. *Int. J. Cancer*, **25**, 197.